# Bridging Them and Us Divisions: A Focus Group Study of Identities in Clinical Psychology Training

**DOI:** 10.1111/tct.70022

**Published:** 2025-01-09

**Authors:** Veenu Gupta, Catrin Eames, Brooke Sharples, Alison Bryant, Beth Greenhill, Laura Golding, Peter Fisher

**Affiliations:** ^1^ Department of Primary Care and Mental Health University of Liverpool Liverpool UK; ^2^ Department of Psychology Durham University Durham UK; ^3^ School of Psychology Liverpool John Moores University Liverpool UK

**Keywords:** clinical psychology, lived experience, personal identity, professional identity, service user involvement

## Abstract

**Background:**

The training of clinical psychologists is conducted by staff, trainees, service users and carers. Often those working in clinical psychology do so due to their own lived experiences. These stakeholders may require having to navigate both personal and professional identities. Whilst there is motivation to make visible their lived experiences, this action may differ dependent on the roles they are in. This study aimed to understand identities within UK clinical psychology training and to enable effective teamworking.

**Method:**

Focus groups were used to socially construct and explore identity constructions of groups in clinical psychology training. The data were thematically analysed using a social constructionist lens.

**Findings:**

Four themes were found. Theme 1 identified ‘dynamics of identity’ where personal and professional identities were ‘integrated’, ‘separated’, ‘permeable’ or ‘visible/invisible’. Theme 2 found the ‘impact of language and labels to rebalance power’, encompassing, ‘expectations and invalidation of a label’ and motivations to ‘rebalance the power’. Theme 3 constructed ‘learner’ and ‘expert’ identities for each group, and Theme 4 found ‘Them & Us divisions’ that speak to the ‘Barriers’, between groups that participants wanted to bridge through modes of ‘Connections’.

**Conclusions:**

This is the first study to use focus groups to socially construct and explore identities in clinical psychology training. The research gives clarity to identities in clinical psychology training, identifying the unique and common ways different stakeholders negotiate professional and personal identities that can promote understanding between stakeholders and better collaboration.

## 1 Background

Key contributors to UK Clinical Psychology training are clinical and academic psychologists. They have the training to perform these roles and are referred to as experts by qualification (EBQ). Other equally important contributors are service users and carers who have experience of disability and of psychological services, or of supporting a loved one who has, referred to as experts by experience (EBE). Both EBQ and EBEs select, teach and evaluate UK trainee clinical psychologists' clinical and research skills. Both these trainers are perceived to be beneficial [1]. EBE support trainees to be person‐centred [1] and enhance learning and clinical skills [2], empathy [3] and critical thinking [4]. Whilst EBE involvement benefits trainee clinical psychologists, the roles also impact their own recovery and identity [5].

Social identity theory suggests we feel more like our in‐group and different to out‐groups [6]. Roles that individuals perform in a structured society are formative to identity [7]. Identities are reinforced through interactions, promoting individuals to behave consistently with the meanings attached to their roles, including the EBE, trainee, carer or EBQ.

Tse et al. [8] suggest EBE involvement leads to changes in social identity. The EBE role encourages professional and politicised identities, moving EBEs beyond their stigmatised patient roles [9]. Identity changes also affect carers due to losing who they were and their new relationship to their loved ones [10]. Mental health professionals must navigate personal and professional identities [11]. Trainee clinical psychologists negotiate learner and practitioner roles [12] and clinical psychologists, practitioner and researcher [13].

A systematic narrative review [14] found the identities of lived experience providers consisted of professional and patient identities that were integrated or unintegrated. Themes in the EMERGES framework influenced this: empowerment, motivation to integrate, empathy of the self and others, recovery model and medical model, growth and transformation, exclusion and survivor roots. Included studies had flaws with limited research on carers and from clinical psychology, providing a rationale to better understand the identities in clinical psychology training, of EBEs, carers, trainee psychologists and EBQs to support greater collaboration.

## 2 Aims

The aims were to understand identities within UK clinical psychology training, of EBEs (service users and carers), trainee clinical psychologists and EBQs using focus groups.

## 3 Methodology

### 3.1 Design

Four separate focus groups were conducted of EBEs, carers, EBQs (clinical/academic psychologists) and trainee clinical psychologists to explore their identities. These groups were mutually exclusive based on how participants self‐defined their occupational roles in UK clinical psychology training.

### 3.2 Inclusion Criteria

Participants had to self‐define as EBEs, carers, trainee clinical psychologists or EBQs (academic or clinical psychologists) working within UK clinical psychology training. They were not limited by demographics and had to be adult age. It was acknowledged individuals may span multiple roles, such as a trainee being an EBE and a clinical psychologist being a carer, but eligibility for joining a focus group was based on their occupational role within clinical psychology training. Where there were dual identities like an EBE also being a carer, they were asked to join the group most salient to them.

### 3.3 Recruitment

Recruitment advertisements were circulated on Twitter, to all UK Doctorate Clinical Psychology programmes, and via British Psychological Society's Group of Trainers in Clinical Psychology network.

### 3.4 Participants

Twenty‐five participants from 9 Doctorate in Clinical Psychology programmes, out of 28 programmes in England, participated. Of the 25, eight were trainee clinical psychologists, six EBEs, seven carers and four EBQs (two Clinical Psychologists and two Academic Psychologists). See Table 1.

**TABLE 1:** Sample characteristics.

Focus group	Sample: *N* = 25	Gender 16 female 9 male	Age 28–75 years	Ethnicity	Context
Trainee psychologist	*N* = 8	7 female 1 male	28–36 years	White, White other, mixed race (White and Caribbean)	In person
Expert by experience	*N* = 6	2 female 4 male	35–65 years 1 participant did not disclose	White, British Cypriot	Online
Expert carer	*N* = 7	5 female 2 male	56–75 years	White and White other	Online
Expert by qualification	*N* = 4	2 female 2 male	37–62 years	White, Latinx/Hispanic	Online

### 3.5 Ethics

University ethical approval was granted (REF ID 5417). Participation was dependent on eligibility and informed consent. Participants were debriefed following each focus group. Data provided were kept confidential and identities pseudo‐anonymised. Participants could withdraw data up to the point of anonymisation. Participants were offered a voucher and travel expenses. Two participants asked for partial data removal, but because the dialogue was socially constructed, these data may have contributed to discourse but were removed prior to analysis.

### 3.6 Positionality

V.G. led the research based on her experiences of positive changes in identity through EBE involvement in UK Clinical Psychology training. The supervisory team included academic research psychologists, c.e., clinical psychologists, P.F., L.G. and B.G., and EBE, A.B., in clinical psychology training. B.S., a psychology student, supported analysis. V.G. kept a reflective diary to reflect on similar or different experiences to her own and was supported with reflexive supervision.

### 3.7 Focus Groups

Focus groups were used to investigate the research question. Touraine [15] believed that working with strangers in a focus group would mirror the social movement they belong to. The focus groups were in person, or via Zoom conferencing services, due to COVID‐19 restrictions. The discussions were audio‐recorded, transcribed and analysed using NVIVO. VG was the interviewer and followed a coproduced semi‐structured interview schedule.

### 3.8 Analysis

A social constructionist approach with critical realism was taken. Thematic analysis was used, which is not bound by epistemology and can be used for multiple datasets [16]. The stages were as follows: (1) The researcher familiarised herself with the data by reading transcripts and listening to recordings, (2) coding the data in an inductive way, (3) generating initial themes, (4) reviewing themes, (5) labelling themes and then (6) defining themes as a research team. Quality of data, no new emerging ideas and team expertise informed data saturation [17]. V.G. analysed each dataset, which was validated by BS's independent analysis of a subset with good agreement, legitimising the findings.

## 4 Results

Four themes were identified. Theme 1 found personal and professional identities, which were ‘integrated’, ‘separated’, ‘permeable’ and ‘visible or invisible’. Theme 2 was the impact of language and labels that led to ‘expectations and invalidation of a label’ with motivations to use language to ‘rebalance the power’. Theme 3 found ‘learner and expert’ identities regarding ability to engage in coproduction. Theme 4 found ‘Them & Us divisions’ through ‘Barriers’ and found ‘Connections’. See Table 2 for summary. See Appendix 1 for extended themes.

**TABLE 2:** Overarching and subordinate themes.

	Themes	Subthemes	Trainee	EBE	Carer	EBQ
1.	Dynamics of Identity	Separation	x		x	x
		Integration	x	x	x	x
		Permeable	x	x	x	x
		Invisible/visible	x	x	x	x
2.	The impact of language and labels to rebalance power	Expectations and invalidation of a label	x	x	x	x
		Rebalance the power	x	x	x	
3.	Learner and expert	Learner	x	x		x
		Expert		x	x	x
4.	Them and us divisions	Barriers	x	x	x	x
		Connections	x	x	x	x

### 4.1 Theme 1: Dynamics of Identity ‘a Strange Mix of Personal and Professional and Caring …’

This theme described personal and professional identities for each stakeholder, which were separated, integrated, permeable or visible or invisible depending on motivations or perceived stigma.

#### 4.1.1 Separation

This subtheme described EBQ, carer and trainees' need for separation between their personal and professional roles to reduce the burden from lived experiences.

Trainee, Serena, wanted to separate her clinical skills from her personal life. ‘I understand why reflective practice is … important … when you're working clinically, but I don't want to be reflective in my personal life … I'm determined for those identities to stay separate …’

I understand why reflective practice is … important … when you are working clinically, but I do not want to be reflective in my personal life … I'm determined for those identities to stay separate …

EBQ, Sharon, an academic with lived experience, found how others drew distinctions between professional and lived experience roles. ‘… they are social constructs … thought of as mutually exclusive …. someone was coming to train us … to do service user involvement and he was making a virtue of … this isn't about professionals and … I felt like that professional bit of me was being pushed out of the room.’

Anthony highlighted how carers are consumed by healthcare issues and sometimes separation is needed from this. ‘… sometimes … everything's around service provision … rather than getting drip fed what was going on with health and social care …, sometimes carers just need that escapism …’

#### 4.1.2 Motivations and Conflicts to Integration

Trainees wanted to integrate their lived experiences but did not know how to, whereas EBEs did this confidently.

Trainee, Ruth, identified how as she grew in her role she wanted to integrate her lived and professional experiences. ‘… much earlier on in my route into training … I wanted to push them separately … whereas now I'm … keen to bring those two things together … and feel like a more coherent … version of me …’

… much earlier on in my route into training … I wanted to push them separately … whereas now I'm … keen to bring those two things together … and feel like a more coherent … version of me …

EBE, Zara, channelled theoretical models she identifies with into teaching. ‘I do quite a lot of teaching … coming from a very much trauma informed (approach) … what you can do is try and embed aspects of that in …, people's day to day working.’

#### 4.1.3 Permeable

The subtheme represented the seeping of the professional role into personal life and vice versa.

Trainee, Mary, who is a trainee clinical psychologist and a peer worker, said her lived and professional experiences were inseparable. ‘… I don't think you can necessarily untangle the two identities.’

… I do not think you can necessarily untangle the two identities.

Carer, Jane, spoke of how the carer role was inextricably linked to those they supported. ‘… it's always the way that if the Carer goes down, the person they care for is always down, so that's two people in hospital.’

Trainee, Ruth, felt there was an expectation to abide by the professional code of conduct in her personal life. ‘… there's a sense of responsibility … you are expected to uphold the professional values in your personal life …’

#### 4.1.4 Invisible/Visible

This subtheme described the visibility of lived experience. Trainees spoke of a need to share experiences in training but felt the space was not safe for this. EBEs articulated the purpose of their roles was to increase visibility. Carers felt their needs were invisible.

Trainee, Jess, found the trainee identity occluded their lived experiences. ‘… we had some trauma teaching, and it was quite distressing … there wasn't anything about looking after yourself in the lecture … the lecturer had almost come in on the assumption … this isn't going to affect any of you …’

EBE, Denise, identified the purpose of the EBE role was to be visible. ‘… sometimes … we're going to be stepping on … people's toes … because we need to be heard … the doctors can't admit to being a service user …’

… sometimes … we are going to be stepping on … people's toes … because we need to be heard … the doctors cannot admit to being a service user …

### 4.2 Theme 2: The Impact of Labels to Rebalance Power ‘in Effect Any Label, It's How It's Used …’

This theme found labels describing groups in clinical psychology training reinforced stereotypes, resulting in ‘expectations and invalidation of the labels’. Participants identified how carefully chosen terms could rebalance power.

#### 4.2.1 Expectations and Invalidation of a Label

Belonging to racial minorities, regional and class identities were discussed across trainees. Some felt they did not fit the typical clinical psychologist stereotype.

Trainee, Jess, identified how others did not construct a clinical psychologist to be mixed race and how the label and its meaning did not recognise her. ‘… I've had service users go, oh is it you that I'm seeing …, people often don't expect … a mixed‐race young woman to turn up.’

EBE, Phil, identified labels chosen for EBEs by others could negatively impact them.

Language and labels have changed over the years … they didn't care how it affected the individual … In effect any label, it's how it's used …, service user, expert, it doesn't matter … you can throw whatever label or term at me …

EBEs and carers agreed that the EBE label was the best option they had, but it did not convey the complexity of their expertise. Carers rejected their label due to stigma.

#### 4.2.2 Rebalance the Power

There were motivations to rebalance power as articulated by trainees, carers wanted more and EBEs wanted to flatten the hierarchy. Language could allay power imbalances.

Trainee, Freya, identified using personal terms to introduce herself could rebalance power. ‘… do you … say, “I'm Dr so and so,” or …, “Just call me (name)?” … those structures give you power … So it's how to rebalance that.’

… do you … say, ‘I'm Dr so and so,’ or …, ‘Just call me (name)?’ … those structures give you power … So it's how to rebalance that.

Carer, Miriam, advocated an alternative term to ‘carer’ to increase status. ‘… we need to come up with a new word … like we are propping up members of society, but no one gives us value.’

### 4.3 Theme 3: Learner and Expert ‘We've All Got Expert Parts and Learner Parts’

This theme identified how groups in clinical psychology training were constructed as learner and expert via juxtaposition. EBEs and carers constructed trainees as learners regarding their abilities to engage in coproduction.

#### 4.3.1 Learner

Trainee clinical psychologists were constructed as ‘learners’ as they were uncritical towards trends in their profession, taking things at face value without critical thought.

EBE, Michael, constructed the trainee as a learner ‘… … their inability to critique their own professional knowledge, if somethings fashionable within their profession at that time, they can't step back …. and see why they … might want to challenge … particularly for people … early on in their career …’

Carer, Miriam, also constructed the trainee as a learner. ‘… they can't imagine that somebody is saying … they're taking medication when they're not … sometimes … trainees have … resistance to the reality.’

#### 4.3.2 Expert

This subtheme described how expert identities were constructed in opposition to others constructed as learners.

Carer, Joy, constructed the expert carer as someone who could identify the needs of carers new to their role. ‘… perhaps it's their first time being there … they are asking questions, but they are not quite asking the question that they want to ask … We know what it is we are looking for … because we have been that person.’

Trainee, Serena, identifies her supervisor as an expert and herself as learner. ‘… my supervisor … can come up with … these amazing suggestions … I know about that theory, and … this theory but why did I not connect them in the same way?’

EBQ, David, constructed trainees as experts to learn from, ‘there's been a lot of furores … around … race, representation, whiteness … saying what … psychological approaches are vs. learning and listening … the generational comment is … important … people coming … on to training … access to very different views. These are powerful forces and we're feeling the effects now. It's going to be good to be part of that … not me as an expert guiding it but seeing how those forces shape it …’

### 4.4 Theme 4: Bridging Them and Us Divisions ‘The Bridge Between Two Different Types of Knowledge’

This theme identified structural barriers and modes of connection between EBE involvement and trainee clinical psychologists.

#### 4.4.1 Barriers

Carer, Louise, noted EBQs were slow to implement changes from EBE. ‘… people are still thinking … we could have … trauma informed care for this service … These are not new ideas … yet they're still not being introduced.’

Trainee, Mary, identified barriers between trainees and EBE, ‘It doesn't feel like there's really open communication … very much … when they're on the timetable, that's when they're available.’

EBQ, Simon, highlights funding as a barrier. ‘… we've got a limited budget … there's only so many (projects) we can fund …. They're not on the same payroll … My guess is (they) don't feel like members of staff … there is an us/them divide still.’

… we have got a limited budget … there's only so many (projects) we can fund …. They're not on the same payroll … My guess is (they) do not feel like members of staff … there is an us/them divide still.

#### 4.4.2 Connections

Carer, Benjamin, articulated how sharing emotion connected groups. ‘If you sort of sprinkle the emotion in there when you're … talking to them, that actually gets through to them … Better than … quoting loads of technicalities.’

EBQ, Sharon, identified service user involvement required negotiation to enable collaboration. ‘… very difficult getting in that them and us space … but we've got a very good coordinator of service user experience who … pours oil on troubled waters and keeps us all … in line … steering that difficult path between how … we get things changed, but … not alienate everyone in the process.’

… very difficult getting in that them and us space … but we have got a very good coordinator of service user experience who … pours oil on troubled waters and keeps us all … in line … steering that difficult path between how … we get things changed, but … not alienate everyone in the process.

## 5 Discussion

This study aimed to understand the identities within UK clinical psychology training. Four themes were found. Theme 1 identified personal and professional identities, sometimes required ‘Separation’, ‘Integration’ and ‘Permeability’ and were either ‘Visible or Invisible’. Theme 2 found labels describing these groups resulted in ‘Expectation and invalidation of a label’, with motivations to ‘Rebalance the power’. Theme 3 found Expert and Learner identities. Theme 4 found ‘Them & Us divisions’ that could be bridged. Findings will be discussed in relation to existing research.

### 5.1 Dynamics of Identity

Participants were asked to self‐define into their roles in clinical psychology training. Becker [18] suggests individuals behave in ways consistent with the meanings attached to the labels describing them, potentially influencing the findings.

EBEs wanted to share their experiences despite its burden [19]. However, separation of identities was important to trainees, so they did not have to remain accountable to their profession in their personal lives and for carers to reduce emotional burden. Llewelyn and Gardner [20] identify boundaries that help maintain distance from service users.

EBEs integrate lived experiences in their work. Some trainees wanted to also be able to do this better [21] and can learn from mental health professionals who integrate lived experiences effectively [13].

Carers felt their needs were invisible as they were understood through the lens of those they supported [12]. EBEs were seen as patients and not professional. Conversely, the EBQ perceived as professional and not patients. Gupta et al. [16] found identity constructions were due to stigma and epistemic injustice. British Psychological Society [22] guidance on valuing lived experience may shift identities in the future.

### 5.2 Learner and Expert

In contrast to EBEs, trainees felt inexperienced at integrating lived experiences. This may be an effect of the ‘trainee’ label, reproducing the ‘learner’, and the EBE, the ‘expert’ [18]. Across groups, expert identities were constructed in opposition to learner identities [23]. Trainees constructed themselves as novice, needing guidance from EBQ [24].

### 5.3 Them and Us

The focus groups may have exacerbated, ‘Them and Us’ divisions [25]. These could be bridged through emotional connections [26] and organisational identification [27].

Trainees identified stereotypes of the clinical psychologist regarding race, gender, class and disability, leading to feelings of unbelonging. Guidance on addressing inequalities could help [28].

### 5.4 Contextual Factors

The study was impacted by COVID‐19 affecting clinical psychology training. Discourse from the Group of Trainers in Clinical Psychology Training Conference [29] and The Black Lives Matter movement may have also influenced the findings.

### 5.5 Strengths and Limitations

This was the first study to explore the collective identities within clinical psychology training. The focus groups may have exacerbated group differences but identified modes of connection. If the focus groups were cross‐disciplinary, it may have resulted in different identity constructions. The study had small samples and lacked diversity, which is symptomatic of this context. Further research is required as future identities may shift due to drives encouraging equity. The small groups meant participants engaged in meaningful dialogue.

## 6 Conclusions

The research identified that EBEs, carers, trainee psychologists and EBQs in clinical psychology training navigated personal and professional identities and barriers between them. These could be bridged through sharing lived experiences, rebalancing power and listening and learning. Common identities found can help connect groups in the field.

## Author Contributions

**Veenu Gupta:** conceptualization, writing – original draft, investigation, methodology, writing – review and editing, formal analysis, project administration. **Catrin Eames:** conceptualization, writing – review and editing, project administration, supervision, formal analysis. **Brooke Sharples:** writing – original draft, methodology, formal analysis. **Alison Bryant:** conceptualization, writing – review and editing, supervision. **Beth Greenhill:** supervision, writing – review and editing, formal analysis, conceptualization. **Laura Golding:** conceptualization, supervision, formal analysis, writing – review and editing. **Peter Fisher:** writing – review and editing, supervision, formal analysis.

## Disclosure

Part of the data for this manuscript are published in a pre‐print.

Veenu Gupta, Catrin Eames, Brooke Sharples et al. A Social Construction of Identities in Clinical Psychology Training in the UK: A Focus Group Study, 23 September 2024, PREPRINT (Version 1) available at Research Square [10.21203/rs.3.rs‐5123678/v1].

## Ethics Statement

The University of Liverpool granted ethical approval for this research.

## Consent

All participants consented to participate in the study and for their data to be used for publications.

## Conflicts of Interest

The authors declare no conflicts of interest.

## Data Availability

The data that support the findings of this study are available on request from the corresponding author and deposited in University of Liverpool repository.

## Appendix 1 Themes Generated With Substantiated Data and Extended Quotes

### 1.1 Theme 1: Dynamics of Identity ‘a Strange Mix of Personal and Professional and Caring’

This theme described how personal and professional identities applied to each stakeholder that requires negotiation. These identities sometimes permeated each other were actively separated or integrated depending on motivations to conceal or reveal lived experiences dependent on perceived stigma

#### 1.1.1 Separation ‘I'm Determined for Those Identities to Stay Separate’

This subtheme described EBQ, carer and trainee participants' need for separation between their personal and professional roles. This separation reduced the burden of lived experience on the individual.

Trainee, Serena, spoke about separating her role as a trainee clinical psychologist and her personal life. ‘I understand why reflective practice is really important … when you're working clinically, but I don't want to be reflective in my personal life … I want to be reflective in my professional role, when I come home … I'm a sister, daughter, partner … I'm determined for those identities to stay separate …’

EBQ, Sharon, discussed how lived and professional experiences were considered as distinct, and felt forced out of the conversation as a professional with lived experience. ‘… they are social constructs that tend to be thought of as mutually exclusive. I mean they're not in reality …. someone was coming to train us about how to do service user involvement and he was making a virtue of … this isn't about professionals and … I felt like that professional bit of me was being pushed out of the room.’

Anthony highlighted how his carer experience was consumed by healthcare issues and how there was a need to separate from this burden. ‘… sometimes … everything's around service provision … rather than getting drip fed what was going on with health and social care …, sometimes carers just need that escapism …’

#### 1.1.2 Motivations and Conflicts to Integration ‘I'm Almost Wanting to Bring Those Two Senses of Self Together’

Trainee participants wanted to integrate their lived experiences into their roles but did not know how to do this, whereas EBE participants were able to do this confidently.

Trainee, Ruth, identified how she wanted to integrate both her lived and professional experiences in her role, which was different to her earlier motivations. ‘… I'm almost wanting to bring those two senses of self together … much earlier on in my route into training … I wanted to push them separately … Whereas now I'm … keen to bring those two things together … and feel like a more coherent … version of me …’

EBE, Zara, spoke of how she sought to integrate models she identifies with into the teaching she does. ‘I do quite a lot of teaching in … universities … coming from a very much trauma informed (approach) … what you can do is try and embed aspects of that in …, people's day to day working.’

EBQ, Maria, said that speaking about her own personal experiences helped reduce her expert status and connect her to EBEs. ‘I felt like because of my expert status they were like … here she comes telling us what to do … I had to really express where I come from more and more … so … they don't see me as the professional.’

#### 1.1.3 Permeable ‘I Don't Think You Can Necessarily Untangle the Two Identities’

The subtheme of permeable identities represented the seeping of the professional role into personal life and vice versa. This was described by trainees and EBQ participants similarly. Carer participants articulated that their own personal identities tied them to those they supported.

Trainee, Mary, who is a trainee clinical psychologist and also worked in lived experience roles, described how her lived and professional experiences were inseparable. ‘… I think it depends on who sees me … I don't think you can necessarily untangle the two identities.’

In a comparable way, carer, Jane, spoke of how the carer role and the health outcomes of those they support are inextricably linked. ‘… it's always the way that if the Carer goes down, the person they care for is always down, so that's two people in hospital.’

Trainee, Ruth, spoke of the HCPC regulations and how there was an expectation to abide by the professional code of conduct in her personal life. ‘… in that profession there's a sense of responsibility … that doesn't just finish when you finish your day … you are expected to uphold the professional values in your personal life …’

#### 1.1.4 Invisible/Visible ‘Sometimes Our Voices Are Just Not Heard and Sometimes … We're Going to be Stepping on … People's Toes … Because We Need to Be Heard … the Doctors Can't Admit to Being a Service User …’

This subtheme described the visibility of lived experience within the profession. Trainee participants spoke of a need to share their lived experiences but felt that clinical psychology spaces did not feel safe for this. EBE participants talked about how the purpose of their roles was to increase visibility. In contrast, carers felt they and their needs were invisible.

Trainee, Jess, spoke of how the trainee identity occluded the visibility of lived experience. ‘I wouldn't describe myself as an expert by experience but … we had some trauma teaching, and it was quite distressing … there wasn't anything about looking after yourself in the lecture … the lecturer had almost come in on the assumption … this isn't going to affect any of you …’

EBE, Denise, identified how the purpose of the EBE role, in contrast to the EBQ role, was to make lived experiences visible. ‘… sometimes our voices are just not heard and sometimes … we're going to be stepping on … people's toes … Because we need to be heard … the professors and the doctors can't admit to being a service user …’

Carer, Miriam, identified how the carer's needs were secondary to all, meaning their needs were not recognised by anyone. ‘… one's own needs can get so suppressed that they're not being recognised, even by the individuals themselves.’

### 1.2 Theme 2: The Impact of Labels to Rebalance Power ‘In Effect, Any Label, It's How It's Used … so You Can Throw Whatever Label or Term at Me …’

This overarching theme described how labels used to describe groups in clinical psychology training reinforced stereotypes resulting in ‘expectations of the labels’ they were understood through and their behaviour in response to this. The labels had an impact on perceived power dynamics across groups. The participants identified how carefully chosen labels could rebalance the power. There was common consensus that labels were invalidating and burdensome

#### 1.2.1 Expectations and Invalidation of a Label ‘… People Often Don't Expect … a Mixed‐Race Young Woman to Turn Up’

This subtheme captured the different perspectives of trainee clinical psychologists, EBEs and carer participants regarding labels used to describe them. It described how labels reinforced stereotypes held by others and could be invalidating based on perceived meanings associated with them.

Belonging to racial minorities, regional and class identities were discussed across trainee participants. Some felt they did not fit the typical clinical psychologist stereotype.

Trainee, Jess, identified how others did not construct a clinical psychologist to be mixed race and this expectation was invalidating. ‘… I've had service users go, oh is it you that I'm seeing, I didn't think you'd look like that …, people often don't expect … a mixed‐race young woman to turn up.’

Trainee, Mary, described how in some ways she does and does not fit the stereotypical clinical psychologist, ‘… it's sometimes the messages, because I'm really conscious that I'm, white, posh, female …. you often hear that you're the perfectionist, you're this, you're the that, which is completely not me at all …’

EBE, Phil, identified labels and language were chosen for EBEs and could negatively or positively impact those being labelled.

Language and labels have changed over the years … they did not care how it affected the individual … In effect any label, it's how it's used … so, service user, expert, it does not matter … you can throw whatever label or term at me …

EBEs concurrently agreed that the EBE label was the best option they had, but it did not convey the complexity of their expertise. For similar reasons, carers rejected their label and also ‘informal carer’ and ‘unpaid carer’.

#### 1.2.2 Weight of a Label ‘… the Word Expert Feels a Bit Pressured …’

This subtheme described how language and labels used to describe groups in clinical psychology training impacted the carer or EBE, due to the power or burden it exerted.

Carer, Joy, described the impact the carer label had on her, replacing her identity of being a mother with an impersonal term. ‘At the beginning being a carer hit me like a ton of bricks … I was a mum and the next thing I have this crisis worker … said I'm the carer. There was no warning … It was like someone had taken my role … as mum, put it in the bin and given me a new title …’

EBQ, Simon, observed the effect of the expert label exerting additional pressure. ‘When I heard the title Experts by Qualification … the word expert feels a bit pressured … I think of Experts by Experience all the time and I've never thought that could … put a lot of pressure on someone …’

#### 1.2.3 Rebalance the Power ‘… Those Structures Give You Power … so It's How to Rebalance That …’

This subtheme identified motivations across each group to rebalance power. Trainee participants felt a need to reduce their power, carer participants wanted to increase this and EBE participants wanted to flatten the hierarchy. The subtheme identified how language and labels could allay power differences.

Trainee, Freya, identified how the label of ‘Doctor’ created a power difference between the patient and the EBQ which could be rebalanced with language. ‘There's a bit of a debate … when you get Doctorate … do you come in and say, “I'm Dr so and so,” or …, “Just call me (name)?” … coming from that standpoint of, we are all equal … I guess … those structures give you power … So it's how to rebalance that.’

Carer, Miriam, advocated for a revaluation of the terms used to describe carers to reduce stigma and increase power. ‘… we need to come up with a new word. That word carer … it's so undervalued … It's got so many negative connotations, like we are propping up members of society, but no one gives us value.’

### 1.3 Theme 3: Learner and Expert ‘… We've All Got Expert Parts and Learning Parts’

This theme identified how groups in clinical psychology training were constructed as both learner and expert in juxtaposition. Trainee participants focused on their own inexperienced identities by comparing themselves to EBQs, constructed as experts. EBEs and carers constructed learner identities of trainee clinical psychologists. All stakeholders were constructed as ‘Learners’ regarding their abilities to engage in effective coproduction.

#### 1.3.1 Learner

This subtheme constructed the learner identity of trainee clinical psychologists as individuals uncritical towards lived experience work or trends in their profession and how they may have taken things at face value without critical thought.

EBE, Michael, constructed the trainee as a learner ‘… … their inability to critique their own professional knowledge, if somethings fashionable within their profession at that time, they can't step back …. and see why they … might want to challenge … I think particularly for people … early on in their career are really precious of that knowledge …’

Carer, Miriam, thought of the trainee as a learner as they did not feel their reality of their lived experiences was understood by them, when they took things at face value. ‘Sometimes, they don't want to hear what you say because it's not what they would expect … they can't imagine that somebody is saying … they're taking medication when they're not, and I find sometimes … trainees have … resistance to the reality.’

#### 1.3.2 Expert

This subtheme described how expert identities were constructed by constructing oppositional groups as learners. The EBQ constructed expert identities of other EBQs as those that listen and learn from others.

Carer, Joy, constructed the expert carer as someone who could spot the needs of other carers new to their role as they had been through the learning process of becoming a carer. ‘… we could see … perhaps it's their first time being there … they are asking questions, but they are not quite asking the question that they want to ask … We know what it is we are looking … because we have been that person.’

Trainee, Serena, viewed her supervisor as an expert by seeing herself as a learner. ‘… my supervisor … can come up with all of these amazing suggestions … I know about that theory, and … this theory but why did I not connect them in the same way … ?’

EBQ, David, suggested the expert EBQ was someone who listened and learned, ‘there's been a lot of furores …. around ethnicity, race, representation, whiteness, and expertise and saying what … psychological approaches are vs. learning and listening … the generational comment is … important … people coming … on to training … increased communication … access to very different views. These are powerful forces and we're feeling the effects now. It's going to be good to be part of that … And not me as an expert guiding it but seeing how those forces shape it …’

### 1.4 Theme 4: Bridging Them and Us Divisions ‘the Bridge Between Two Different Types of Knowledge’

This overarching theme identified how EBEs, carers, trainees and EBQs felt more like their in‐group and different to others. Sometimes, they also felt different to their own social group, influenced by stances on mental health. Each group felt barriers to other groups and wanted to find ways to connect.

#### 1.4.1 Feeling Similar and Different ‘I Feel Most Connected to People Who Move Beyond an Individual Experience and Turn It Into a Collective Change’

This theme described how each member of the focus groups felt similar but also different to members in the group, with shared goals and motivations to support their group.

EBE, Zara, said she related to EBEs who wanted to make a difference for the group. ‘I feel most connected to people who move beyond an individual experience and turn it into a collective change.’

EBE, Michael, connected better with EBEs who understood their mental health experiences similarly to him, ‘… if someone's promoting a sort of purely medical perspective, I find that quite difficult to connect with ….’

EBQ, Sharon, highlighted her feelings of difference with other EBQs as a research psychologist with lived experience. ‘… at first feeling they are all pussy footing around me, is it because I'd said to occupational health about my mental health … I thought it's because I'm a researcher, they are thinking … she's going to discover we are crap at stats ….’

#### 1.4.2 Barriers and Connections ‘… Very Difficult … Getting in That Them and Us Space … but We've Got a Very Good Coordinator of Service User Experience Who … Pours Oil on Troubled Waters and Keeps Us All … in Line … Steering That Difficult Path Between How … We Get Things Changed, but … Not Alienate Everyone in the Process’

This subtheme described how there were barriers to meaningful lived experience work created by EBQs with motivations to connect groups.

Carer, Louise, noted how EBQs were slow to implement changes from her input. ‘What worries me is in our involvement … people are still thinking … we could have … trauma informed care for this service … These are not new ideas … yet they're still not being introduced.’

There was a consistent narrative from trainee participants of their disconnection with EBEs. Trainee, Mary, said, ‘it doesn't feel like there's really open communication … it feels very much that it's led by when they're on the timetable, that's when they're available.’

EBQ, Simon, highlighted the structural barriers EBEs experienced. ‘… we've got a limited budget … there's only so many (projects) … we can fund … they're never going to feel equal. They're not on the same payroll, they're not embedded into the structure on our course … My guess is (they) don't feel like members of staff … there is an us/them divide still.’

The theme highlighted how to negotiate and bridge connections between groups. Carer, Benjamin, articulated the power of emotion in enabling this. ‘If you sort of sprinkle the emotion in there when you're … talking to them, that actually gets through to them … Better than … just quoting loads of technicalities.’

EBQ, Sharon, suggested that service user involvement required negotiation. ‘… … It's very difficult getting in that them and us space … but we've got a very good coordinator of service user experience who … pours oil on troubled waters and keeps us all … in line … steering that difficult path between how … we get things changed, but … not alienate everyone in the process.’
